# High replacement of soybean meal by different types of rapeseed meal is detrimental to rainbow trout (*Oncorhynchus mykiss*) growth, antioxidant capacity, non-specific immunity and *Aeromonas hydrophila* tolerance

**DOI:** 10.3389/fnut.2024.1363411

**Published:** 2024-02-06

**Authors:** Wen Jiang, Hengzhi Wang, Lu Zhang, Haifeng Mi, Junming Deng

**Affiliations:** ^1^College of Fisheries, Guangdong Ocean University, Zhanjiang, China; ^2^College of Animal Science and Technology, Yunnan Agricultural University, Kunming, China; ^3^Tongwei Agricultural Development Co., Ltd., Chengdu, China

**Keywords:** rapeseed meal, digestive enzyme, intestinal morphology, glucosinolates, *Oncorhynchus mykiss*

## Abstract

A 12-week feeding trial was conducted to evaluate the effects of replacing soybean meal with different types of rapeseed meal (RSM; Chinese 95-type (oil press model) rapeseed meal [C95RM], Chinese 200-type rapeseed meal [C200RM], cold pressed rapeseed cake [CPRC], Indian rapeseed meal [IRM] and Canadian rapeseed meal [CRM]) on growth, antioxidant capacity, non-specific immunity and *Aeromonas hydrophila* infection tolerance in 990 fingering (average weight 12.77 ± 0.01 g) rainbow trout (*Oncorhynchus mykiss*). A basal diet was prepared using fishmeal and soybean meal as the main protein sources, the other 10 diets were formulated with five types of RSM at 20% (C95RM20, C200RM20, CPRC20, IRM20, CRM20) or 35% (C95RM35, C200RM35, CPRC35, IRM35, CRM35) inclusion levels to replace iso-nitrogenous soybean meal. Regardless of the RSM source, dietary inclusion of 20% RSM significantly reduced the weight gain rate (WGR) and digestive enzymes activities (except C200RM20) of fish, but increased the blood urea nitrogen (BUN) and hepatic malondialdehyde (MDA) content (except CRM20). Fish fed with CPRC20 and IRM20 exhibited relatively higher plasma cortisol and MDA content, but lower content/activities of triiodothyronine (T_3_), thyroxine (T_4_) and glutathione peroxidase (GPx) in plasma, lysozyme (LZM) and complement 3 (C3) in serum, catalase (CAT) in liver, and respiratory burst activity (RBA) of head kidney macrophages. The intestinal and hepatic tissues fed with 20% RSM were damaged to some extent, with the CPRC20 and IRM20 groups being the most severely affected. Regardless of the RSM source, dietary inclusion of 35% RSM significantly decreased WGR and digestive enzymes activities, but significantly increased plasma BUN and MDA content. The fish fed with CPRC35 and IRM35 exhibited relatively higher plasma cortisol, MDA, serum triglyceride, BUN content, but lower content/activities of T_3_, T_4_, C3, and LZM in serum, CAT, peroxidase and GPx in plasma, CAT in liver, RBA and phagocytic activity of head kidney macrophage. The hepatic and intestinal tissues damage was the worst in the IRM35 group among the 35% RSM inclusion groups. These results indicate that including ≥20% RSM in the diet, regardless of the source, reduced the growth, antioxidant capacity, immunity, and survival to *Aeromonas hydrophila* infection in rainbow trout.

## 1 Introduction

The unstable supply and high price of conventional protein sources including fishmeal and soybean meal (SBM) have forced the aquaculture industry to seek alternative protein sources. The use of plant-derived protein sources with stable supply and low price to replace SBM or even fishmeal has become a trend (1, 2). Rapeseed meal (RSM) is a high-quality plant-derived protein source with relatively high protein (35–45%) content and balanced amino acid profile (3, 4). In recent years, the production of rapeseed meal has gradually increased, making it the second most widely traded protein component after soybean meal (5). From 2016 to 2020, the global RSM production continued to grow, and which was 40.74 million tons in 2020 (6). However, the quality of RSM mainly depend on the variety, origin and processing method (7, 8), and which is strongly related to the presence of various antinutritional factors such as glucosinolates, tannin, phytic acid and erucic acid levels (9–11). Currently, RSM is primarily produced in European Union, Canada, China, and India. According to the processing method, Chinese RSM is divided into Chinese 95-type RSM (C95RM), Chinese 200-type RSM (C200RM), cold pressed rapeseed cake (CPRC) and mixed type RSM. Among these RSM, Indian RSM (IRM) and CPRC contained relatively higher levels of isothiocyanates, oxazolidinethiones and their degradation products (12, 13). However, the content of antinutritional factors in C95RM, C200RM and Canadian RSM (CRM) were relatively low (14). Studies on black carp (*Mylopharyngodon piceus*), grass carp, gible carp (*Carassius auratus gibelio*), Atlantic salmon (*Salmo salar*), and rainbow trout have shown that excessive anti-nutritional factors in RSM can cause varying degrees of damage to immune, digestive, and physiological functions, and inhibit growth (15). Among them, glucosinolates can impair liver function and cause thyroid follicular cells to become smaller, irregular in shape, with nuclear enlargement, increased mitosis, and other related phenomena. In addition, a concentration of 3 to 6% of sinapic acid can lead to mortality and pathological changes in the skin, gills, kidneys, and heart of silver salmon (16).

Previous studies showed that the addition of less than 30% C200RM or CRM has no negative effect on the growth of tilapia (*Oreochromis niloticus × O. aureus*), while which was depressed by the addition of 30% IRM (17). IRM can replace 15% C200RM in diets without compromising growth performance of common carp (*Cyprinus carpio*) (18). The inclusion level of IRM in grass carp (*Ctenopharyngodon idella*) diet should be less than 20%, while the level of CPRC and CRM addition should be controlled at 20–35% (14). These studies have shown that the effects of RSM vary widely from different types and levels of RSM.

Rainbow trout (*Oncorhynchus mykiss*) is among the highly economical species cultivated all around the world (19). Although RSM is widely used in aquafeed, most studies have focused on the comparisons of single-variety, multi-level or single-level, multi-variety, whereas limited studies were performed based on multi-variety and multi-level of RSM (11, 20). The aim of the present study was to assess the effects of replacing SBM with different types of RSM on the growth performance, digestive enzyme activity, antioxidant capacity, and immune and tissue structure of rainbow trout.

## 2 Materials and methods

### 2.1 Experimental diet

In this study, five RSM (C95RM, C200RM, CPRC, IRM, and CRM) with varying origins or processing methods were used to replace SBM in rainbow trout diets. The nutritional compositions and antinutritional factors (ANFs) content of various RSM were listed in Table 1. Eleven isonitrogenous and isoenergetic (41% crude protein, 22 MJ/kg gross energy) diets were formulated to contain various types and levels of RSM. A basal diet was prepared using fishmeal and soybean meal as the main protein sources, the other 10 diets were formulated with five types of RSM at 20% (C95RM20, C200RM20, CPRC20, IRM20, CRM20) or 35% (C95RM35, C200RM35, CPRC35, IRM35, CRM35) inclusion levels to replace isonitrogenous SBM in the basal diet, respectively. The decision to use the 20 and 35% replacement levels was made for two primary reasons. First, adding 20 or 30% RSM to the rainbow trout diet had no adverse effect on their growth. Second, IRM, CPRC, C95RM, C200RM, and CRM can be added to grass carp or carp feed up to 35% without adverse effects (14). The ingredients, proximate composition and ANFs of the experimental diets are presented in Table 2.

**Table 1:** Nutritional composition and anti-nutritional factors content of different types of rapeseed meal.

	C95RM	C200RM	CPRC	IRM	CRM
**Proximate composition (%)**					
Dry matter	90.04	88.42	91.40	91.00	90.98
Crude protein	37.65	35.86	34.16	39.10	36.41
Crude lipid	0.73	1.52	8.52	0.60	1.49
Crude Ash	7.80	7.20	6.62	7.20	7.20
Calcium	0.75	0.77	0.68	0.31	0.81
Phosphorus	1.10	1.06	1.07	1.06	1.03
**Antinutritional factor level**					
Glucosinolates (%)	0.60	0.97	6.56	4.93	ND
Isothiocyanate (mg/g)	0.53	0.76	1.29	13.53	0.15
Oxazolidinethione (mg/g)	0.05	0.42	3.08	4.70	0.40
Tannins (mg/g)	4.11	4.24	4.72	4.81	1.30
Phytic acid (mg/g)	8.45	6.95	6.35	6.68	0.52
Sinapine (mg/g)	2.55	2.53	2.59	2.88	1.80
Sinapic acid (%)	0.06	0.09	1.64	0.34	0.06
**Amino acid composition (%)**					
Aspartic acid	1.96	2.07	2.05	2.26	2.09
Glutamic acid	5.06	4.98	4.85	6.50	5.11
Serine	1.06	1.05	1.05	1.41	1.09
Glycine	0.99	0.97	0.93	1.66	0.96
Histidine	0.90	0.88	0.87	0.93	0.91
Arginine	1.49	1.65	1.70	2.11	1.75
Threonine	1.76	1.61	1.57	1.36	1.55
Alanine	1.56	1.55	1.44	1.53	1.49
Proline	4.00	3.92	3.37	2.24	3.79
Tyrosine	0.74	0.75	0.75	0.75	0.73
Valine	1.90	1.87	1.77	1.53	1.81
Methionine	0.20	0.18	0.19	0.46	0.18
Cysteine	0.05	0.04	0.04	0.04	0.04
Isoleucine	1.36	1.35	1.28	1.16	1.31
Leucine	2.44	2.40	2.28	2.25	2.31
Phenylalanine	1.19	1.17	1.10	1.44	1.14
Lysine	0.95	1.58	1.79	1.66	1.70

Tryptophan was not detected; ND, not detected. C95RM, Chinese 95-type rapeseed meal; C200RM, Chinese 200-type rapeseed meal; CPRC, cold pressed rapeseed cake; IRM, Indian rapeseed meal; CRM, Canadian rapeseed meal.

**Table 2:** Ingredients and nutritional composition (% dry matter) of the experimental diets.

Ingredient	Control	20% inclusion level of RSM					35% inclusion level of RSM				
		C95RM20	C200RM20	CPRC20	IRM20	CRM20	C95RM35	C200RM35	CPRC35	IRM35	CRM35
Fish meal	32.00	32.00	32.00	32.00	32.00	32.00	32.00	32.00	32.00	32.00	32.00
Soybean meal	40.00	21.00	21.00	22.00	21.00	21.00	8.00	8.00	10.00	8.00	8.00
Wheat flour	2.36	4.42	6.45	7.96	4.42	7.33	1.34	4.91	6.62	1.34	6.43
Wheat starch	9.03	5.25	3.55	2.61	4.93	2.61	5.80	2.81	1.99	5.24	1.19
C95RM	–	20.00	–	–	–	–	35.00	–	–	–	–
C200RM	–	–	20.00	–	–	–	–	35.00	–	–	–
CPRC	–	–	–	20.00	–	–	–	–	35.00	–	–
IRM	–	–	–	–	20.00	–	–	–	–	35.00	–
CRM	–	–	–	–	–	20.00	–	–	–	–	35.00
Leucine	0.43	0.59	0.60	0.63	0.59	0.50	0.67	0.69	0.66	0.67	0.51
Lysine	0.00	0.28	0.15	0.11	0.28	0.34	0.51	0.29	0.16	0.51	0.61
Methionine	0.00	0.11	0.11	0.11	0.11	0.05	0.16	0.16	0.15	0.16	0.05
Fish oil	5.00	5.00	5.00	5.00	5.00	5.00	5.00	5.00	5.00	5.00	5.00
Soybean oil	8.33	8.50	8.29	6.73	8.50	8.32	8.67	8.29	5.57	8.67	8.36
Soybean lecithin	0.50	0.50	0.50	0.50	0.50	0.50	0.50	0.50	0.50	0.50	0.50
Ca(H_2_PO_4_)_2_	1.00	1.00	1.00	1.00	1.00	1.00	1.00	1.00	1.00	1.00	1.00
Choline chloride (50%)	0.30	0.30	0.30	0.30	0.30	0.30	0.30	0.30	0.30	0.30	0.30
Ethoxyquin (30%)	0.03	0.03	0.03	0.03	0.03	0.03	0.03	0.03	0.03	0.03	0.03
Vitamin C	0.02	0.02	0.02	0.02	0.02	0.02	0.02	0.02	0.02	0.02	0.02
Mineral premixes^a^	0.60	0.60	0.60	0.60	0.60	0.60	0.60	0.60	0.60	0.60	0.60
Vitamin premixes^b^	0.40	0.40	0.40	0.40	0.40	0.40	0.40	0.40	0.40	0.40	0.40
**Proximate composition**											
Dry matter (DM, %)	89.91	89.06	90.22	90.45	89.93	89.27	90.46	90.15	90.64	90.65	90.56
Crude protein (% DM)	41.01	41.65	41.43	41.01	41.26	40.98	41.11	41.54	41.06	41.32	41.22
Crude lipid (% DM)	17.22	17.81	17.39	17.16	17.12	17.20	17.68	17.67	17.61	17.19	17.14
Crude Ash (% DM)	9.38	9.67	9.69	10.34	9.49	9.31	10.33	9.88	11.21	11.28	9.49
Energy (MJ/kg)	21.42	22.22	21.90	21.94	22.19	21.52	22.11	22.04	22.01	21.84	21.90
**Antinutritional factor level**											
Glucosinolates (%)	0.00	0.12	0.19	1.31	0.12	0.00	0.21	0.34	2.30	0.21	0.00
Isothiocyanate (mg/g)	0.03	0.11	0.15	0.26	0.37	0.03	0.19	0.27	0.45	0.65	0.05
Oxazolidinethione (mg/g)	0.01	0.01	0.08	0.62	0.01	0.08	0.02	0.15	1.08	0.02	0.14
Tannins (mg/g)	0.29	0.82	0.85	0.94	0.82	0.26	1.44	1.48	1.65	1.44	0.46
Phytic acid (mg/g)	0.20	1.69	1.39	1.27	1.69	0.10	2.96	2.43	2.22	2.96	0.18
Sinapine (mg/g)	0.13	0.51	0.51	0.52	0.51	0.36	0.89	0.89	0.91	0.89	0.63
Sinapic acid (%)	0.01	0.01	0.02	0.33	0.07	0.01	0.02	0.03	0.57	0.11	0.02

a Mineral premix (mg/kg diet): MgSO_4_•7H_2_O, 900 mg; KI, 5 mg; FeSO_4_•H_2_O, 1,300 mg; ZnSO_4_•H_2_O, 900 mg; CuSO_4_•5H_2_O, 125 mg; Na_2_Se_2_O_3_, 0.05 mg; MnSO_4_•H_2_O, 900 mg; CoCl_2_•6H_2_O, 3.75 mg.

b Vitamin premix (mg/kg diet): retinyl acetate (2,800,000 IU/g), 10 g; cholecalciferol, 0.15 g; DL-α- tocopheryl acetate, 150 g; menadione, 15 g; thiamine hydrochloride, 40 g; riboflavin, 55 g; pyridoxine hydrochloride, 40 g; vitamin B_12_, 0.1 g; ascorbic acid, 250 g; folic acid, 5 g; biotin 0.5 g; niacin, 150 g; calcium D-pantothenate, 160 g; inositol, 125 g. C95RM, Chinese 95-type rapeseed meal; C200RM, Chinese 200-type rapeseed meal; CPRC, cold pressed rapeseed cake; IRM, Indian rapeseed meal; CRM, Canadian rapeseed meal. All feed materials are provided by Foshan Nanhai Tongwei Aquatic Technology Co., LTD (Foshan, China).

The experimental ingredients (except for fish oil, soybean oil, and soybean lecithin) were ground through a 320-μm mesh sieve. Then the crushed ingredients were thoroughly mixed with fish oil, soybean oil and soybean lecithin (pre-dissolved in soybean oil), and the mixture was pelleted by an experimental pellet feed mill (KS-180; Jiangsu Jingu Rice Mill Co., Ltd., Jiangsu, China) through a 2-mm diameter die. The moist pellets were dried in a forced air oven at room temperature for about 12 h, and then stored at −20°C until used.

### 2.2 Feeding management

Fingering rainbow trout were temporarily fed (commercial feed, 40% curde protein, 10% curde lipid) in net cages for 2 weeks to acclimatize to the experimental environment. Nine hundreds ninety uniformly sized, healthy fingering rainbow trout (12.77 ± 0.01 g) were assigned at random to 33 net cages (11 groups, 3 replicates per group) with 30 fish per net cage (0.7 m × 0.7 m × 1.0 m). All fish were hand-fed twice daily (07:00 and 17:00) to satiation. During the 12-week feeding trial, the water is recirculated through a biological and mechanical filtration system with continuous oxygenation, natural light, water temperature 16–18°C (during the feeding trial, the water temperature was maintained at 14–16°C for only 2 days), pH 7.5–7.9, dissolved oxygen concentration ≥7 mg/L, ammonia nitrogen concentration 0.04–0.07 mg/L.

### 2.3 Sample collection

At the end of the experiment, all fish were fasted for 24 h. Fish from each net cage were weighed and counted to calculate the survival rate, growth performance and feed utilization. All fish were anesthetized with eugenol (88.9 mg/L) prior to sample collection. Serum (1 fish) or plasma (2 fish) was collected from the caudal vein using a syringe (1 mL, sterile disposable syringe) or a syringe moistened with sodium heparin. All blood samples were allowed to stand for 4 h in a refrigerator at 4°C, then centrifuged at 4°C at 4,000 rpm/min, and the supernatant was stored in a refrigerator at −80°C. After the blood was collected, three fish were dissected. Two liver segments, two foregut segments, and one gill segment, each approximately the size of a green bean, were cut. One liver, foregut, and gill were preserved in 4% formaldehyde fixative, while the other liver and foregut were preserved in a glutaraldehyde solution for subsequent tissue sectioning. The stomach, remaining foregut, and liver were then removed and rapidly stored at −80°C for subsequent determination of relevant enzyme activities. Additionally, head kidney macrophages were isolated according to the method of Secombes (21). Two fish were randomly selected from each net cage and stored in a refrigerator at −20°C for body composition analysis.

### 2.4 Analysis

#### 2.4.1 Chemical composition

The assay determination of feed ingredients, experimental diets and fish body compositions were referred to the method of AOAC (22): moisture was determined by drying at 105°C to constant weight; crude protein and crude lipid were determined by the Kjeldahl (JK9830; Jinan Precision Scientific Instruments & Instruments Co., Ltd., Jinan, China) and Soxhlet (using petroleum ether as solvent) method, respectively; crude ash was determined by sintering at 550°C (16 h, box type resistance furnace SX-410; Beijing Ever Bright Medical Treatment Instruments Co., Ltd., Beijing, China). Total energy was measured by an oxygen bomb energy meter (ZDHW-6; Hebi Huatai Electronics Co., Ltd., Henan, China). The content of calcium and phosphorus were determined by kit (Nanjing Jiancheng Bioengineering Institute, Nanjing, China). The amino acid composition of RSM and experimental diets was determined by the Venusil AA amino acid analysis method (acid hydrolysis) using high-performance liquid chromatography (LC-20AT; Shimadzu, Japan). Glucosinolates, isothiocyanates, oxazolidinethione, tannin, phytic acid, sinapine, and sinapic acid content were analyzed using the method of Yuan et al. (15).

#### 2.4.2 Digestive enzyme activity

Pepsin as well as intestinal lipase, α-amylase, disaccharidase and pepsin were determined using a commercial kit (No. A054-2-1, No. C016-1-1, No. A082-1-1, No. A080-1-1; Nanjing Jiancheng Bioengineering Institute, Nanjing, China), following the instructions.

#### 2.4.3 Blood biochemical index

Triiodothyronine (T_3_), thyroxine (T_4_), total cholesterol (TC), triglyceride (TG), blood urea nitrogen (BUN), alkaline phosphatase (AKP), aspartate aminotransferase (AST), alanine aminotransferase (ALT), total protein (TP), glucose (GLU), lysozyme (LZM), immunoglobulin M (IgM), complement 3 (C3), and complement 4 (C4) were measured using kits (No. H222-1-2, No. H223-1-2, No. A111-1-1, No. A110-1-1, No. C013-2-1, No. A059-2-2, No. C010-2-1, No. C009-2-1, No. A045-1-1, No. A154-2-1, No. A050-1-1, No. H109-1-2, No. H186-1-2, No. H186-2-2; Nanjing Jiancheng Bioengineering Institute, Nanjing, China) according to the methods of Yuan et al. (15). Respiratory burst activity (RBA) and phagocytic activity (PA) of head kidney macrophages were determined by the methods of Secombe (21) and Zhou (23), respectively.

#### 2.4.4 Antioxidant-related parameters in liver

About 0.2 g of liver samples and 1.8 mL iced saline solution were weighted, and homogenized in an ice bath for 20 s (FluKO homogenizer; Shanghai Fluke Fluid Machinery Manufacturing Co., Ltd.), and centrifuged at 2,500–3,500 rpm/min (GTR16-2 freezing centrifuge, Beijing Times Beili Centrifuge Co., Ltd.) for 10 min, and the supernatant was aspirated to determine the antioxidant-related parameters. Glutathione reductase (GR), superoxide dismutase (SOD), Malondialdehyde (MDA), peroxidase (POD), glutathione peroxidase (GPx) and catalase (CAT) were measured using kits (No. A006-1-1, No. A001-1-2, No. A003-1-2, No. A084-2-1, No. A005-1-2, No. A007-1-1; Nanjing Jiancheng Bioengineering Institute, Nanjing, China).

#### 2.4.5 Tissue section

Tissue samples (liver, intestine and gill) were dewaxed, embedded, sectioned, unfolded, baked, stained with hematoxylin–eosin (H&E), and sealed with neutral gel to obtain tissue sections. Microscopic photographs were taken, and relevant sections were collected and analyzed using the Lecia Applied Staining Imaging System. Tissue samples were fixed by glutaraldehyde, post-fixed, dehydrated, dried, and the samples were treated for electrical conductivity to obtain electron microscopic sections, which were observed under a scanning electron microscope for photographs.

#### 2.4.6 Challenge test

Challenge test was performed at 12-week of the feeding trial. *A. hydrophila* strain from the Marine Culture Collection of China (MCCC, 1A00007). Activated *Aeromonas hydrophila* was eluted with saline at a volume fraction of 0.65% to remove the bacterial moss. Forty fish were randomly selected from each group and each fish was injected intraperitoneally with 0.1 mL of live *A. hydrophila* solution at a concentration of 1.0 × 10^8^ cfu/mL (determined by pre-experimentation of semi-lethal infection with spare fish from the same batch), and observed for 1 week, the relative percentage survival (RPS) was calculated: [1– (% mortality in fish fed diet with RSM/% mortality in control)] × 100. Inoculation of dead fish foci confirmed that dead fish were infected with *A. hydrophila*.

### 2.5 Calculation and statistical analysis

The results of all tests are expressed as mean ± standard error (*n* = 3). All statistical analyses were performed using SPSS 17.0 software with one-way analysis of variance (ANOVA) and multiple comparison analysis using Duncan’s test when there was a significant difference (*p* < 0.05) among the dietary treatments. Additionally, Chi-square test was used to assess the difference in RPS of rainbow trout after infection with *A. hydrophila*.

## 3 Results

### 3.1 Proximate composition and anti-nutrition factors of RSM

The five kinds of RSM had similar crude protein (34.16–39.10%) and crude ash (6.62–7.80%) content, but the crude lipid content varied greatly (0.60–8.52%). Compared to the other RSM, IRM had the relatively lower threonine, proline, valine and isoleucine content, but relatively higher levels of aspartic, glutamic, serine, methionine and glycine. Additionally, the CPRC and IRM had relatively higher content of glucosinolates (6.56, 4.93%), sinapic acid (1.64, 0.34%), isothiocyanate (1.29 mg/g, 13.53 mg/g) and oxazolidinethione (3.08 mg/g, 4.70 mg/g) than the other RSM (C95RM, C200RM, and CRM). The sinapine content (2.88 mg/g) of IRM was also relatively higher than the other RSM. On the contrary, the CRM had relatively lower levels of isothiocyanate, tannins, phytic acid and sinapine than the other RSM.

### 3.2 Growth performance

After 12-week feeding trial, the survival rate of fish ranged from 60.00 to 98.67%, which was significantly lower in the IRM20 (61.33%) and IRM35 (60.00%) groups compared to the other groups (*p* < 0.05, Table 3). Dietary 20% RSM inclusion regardless of sources significantly depressed the FBW, WGR and DGC of fish, and those were the lowest in the CPRC20 group (*p* < 0.05). Similarly, the inclusion of 20% RSM generally depressed the PER, but no significant difference was observed between the C200RM20 or CRM20 groups and the control group (*p* > 0.05). Conversely, dietary 20% RSM inclusion generally increased the FCR, but significant difference was only observed between the IRM20 group and the control group (*p* < 0.05). Dietary 20% RSM inclusion did not significantly affect the VSI and HSI of fish (*p* > 0.05), whereas the HSI was significantly higher in the IRM20 group compared to the CRM20 group (*p* < 0.05).

**Table 3:** Growth performance and feed utilization of rainbow trout fed diets with different types and levels of rapeseed meal.

Diets	FBW (g)	FI (g/kg MWB/d)^a^	WGR^b^	DGC (%/day)^c^	FCR^d^	PER^e^	SR (%)^f^	VSI (%)^g^	HSI (%)^h^
Control	94.91 ± 0.79^e^	10.57 ± 0.26^abc^	6.28 ± 0.04^e^	2.55 ± 0.02^e^	1.19 ± 0.04^a^	2.05 ± 0.06^c^	97.33 ± 1.33^b^	4.10 ± 0.13^a^	0.90 ± 0.03^ab^
C95RM20	80.60 ± 1.83^d^	11.35 ± 0.35^abc^	5.30 ± 0.15^d^	2.30 ± 0.04^d^	1.50 ± 0.06^a^	1.59 ± 0.06^b^	88.00 ± 0.00^b^	4.40 ± 0.13^abc^	0.88 ± 0.04^a^
C200RM20	78.00 ± 1.88^d^	10.63 ± 0.60^abc^	5.12 ± 0.14^d^	2.25 ± 0.04^cd^	1.35 ± 0.09^a^	1.80 ± 0.12^bc^	94.67 ± 3.53^b^	4.29 ± 0.09^ab^	0.87 ± 0.04^ab^
CPRC20	54.80 ± 1.07^b^	9.95 ± 0.27^ab^	3.29 ± 0.09^b^	1.70 ± 0.03^b^	1.61 ± 0.06^a^	1.51 ± 0.07^b^	94.67 ± 2.67^b^	4.39 ± 0.16^ab^	1.04 ± 0.05^ab^
IRM20	67.69 ± 2.59^c^	12.78 ± 0.91^c^	4.30 ± 0.20^c^	2.02 ± 0.06^c^	2.67 ± 0.21^b^	0.92 ± 0.07^a^	61.33 ± 5.81^a^	4.39 ± 0.15^ab^	1.11 ± 0.12^bc^
CRM20	79.06 ± 0.73^d^	10.45 ± 0.36^abc^	5.19 ± 0.08^d^	2.27 ± 0.02^d^	1.31 ± 0.04^a^	1.87 ± 0.06^bc^	96.00 ± 0.00^b^	4.39 ± 0.11^ab^	0.85 ± 0.03^a^
C95RM35	73.18 ± 2.06^cd^	10.75 ± 0.37^abc^	4.74 ± 0.18^cd^	2.15 ± 0.05^cd^	1.51 ± 0.08^a^	1.60 ± 0.08^b^	88.00 ± 4.00^b^	4.48 ± 0.12^abc^	0.96 ± 0.05^ab^
C200RM35	72.85 ± 0.79^cd^	10.16 ± 0.49^ab^	4.69 ± 0.07^cd^	2.14 ± 0.02^cd^	1.38 ± 0.04^a^	1.75 ± 0.05^bc^	92.00 ± 2.31^b^	4.79 ± 0.16^bc^	0.99 ± 0.05^ab^
CPRC35	50.95 ± 1.34^ab^	8.86 ± 0.23^a^	3.00 ± 0.11^ab^	1.59 ± 0.04^ab^	1.56 ± 0.07^a^	1.57 ± 0.07^b^	92.00 ± 4.62^b^	5.08 ± 0.19^c^	1.33 ± 0.05^c^
IRM35	45.00 ± 3.29^a^	12.19 ± 0.60^bc^	2.52 ± 0.27^a^	1.41 ± 0.10^a^	4.12 ± 0.41^c^	0.60 ± 0.07^a^	60.00 ± 4.62^a^	4.73 ± 0.23^abc^	1.05 ± 0.05^ab^
CRM35	74.74 ± 1.63^cd^	10.67 ± 0.57^abc^	4.85 ± 0.12^cd^	2.18 ± 0.03^cd^	1.36 ± 0.08^a^	1.80 ± 0.11^bc^	98.67 ± 1.33^b^	4.22 ± 0.12^ab^	0.88 ± 0.03^ab^

Values are means ± SEM (*n* = 3). Means in the same row with different superscripts are significantly different from each other (*p* < 0.05). FBW, final body weight; MBW, mean metabolic body weight = ((initial weight/1,000)^0.75^ + (final weight/1,000)^0.75^)/2.

a Feed intake (FI, g/kg MBW/d) = 100 × dry feed weight (g)/MBW/experimental period.

b Weight gain rate (WGR) = (final weight – initial weight)/ initial weight.

c Daily gain coefficient (DGC, %/d) = 100 × (final weight ^1/3^– initial weight ^1/3^)/experimental period.

d Feed conversion ratio (FCR) = dry feed weight (g)/(final weight – initial weight).

e Protein efficiency ratio (PER) = (final weight – initial weight)/feed protein intake (g).

f Survival rate (SR, %) = 100 × final number of fish/ initial number of fish.

g Viscerosomatic index (VSI, %) = 100 × visceral weight (g)/body weight (g).

h Hepatosomatic index (HSI, %) = 100 × liver weight (g)/body weight (g).

Dietary 35% RSM inclusion regardless of sources significantly depressed the FBW, WGR and DGC, and those were the lowest in the IRM35 group (*p* < 0.05, Table 3). Similarly, the inclusion of 35% RSM generally depressed the PER, but no significant difference was observed between the C200RM35 or CRM35 groups and the control group (*p* > 0.05). Conversely, dietary 35% RSM inclusion generally increased the FCR, but significant difference was only observed between the IRM35 group and the control group (*p* < 0.05). Dietary 35% RSM inclusion generally increased the VSI and HSI, and the highest values were observed in the CPRC35 group.

With the increase of RSM inclusion level, the growth performance (FBW, WGR and DGC) and feed utilization (PER) exhibited a descending trend and the FCR showed an opposite trend, but significant differences were only observed among the IRM groups (*p* < 0.05, Table 3). However, the VSI and HSI were significantly higher in the CPRC35 group compared to the CPRC20 group (*p* < 0.05).

### 3.3 Proximate composition of whole-body of fish

Dietary inclusion of 20% RSM did not affect the whole-body moisture, crude protein, crude lipid, crude ash and total energy content of rainbow trout (*p* > 0.05, Table 4).

**Table 4:** The whole-body composition of rainbow trout fed diets with different types and levels of rapeseed meal.

Diets	Moisture (%)	Crude protein (%)	Crude lipid (%)	Crude ash (%)	Gross energy (MJ/kg)
Control	62.80 ± 0.61^a^	17.39 ± 0.59^b^	15.64 ± 0.59^cd^	2.64 ± 0.01	10.51 ± 0.19^d^
C95RM20	64.15 ± 1.05^ab^	16.33 ± 0.88^ab^	16.15 ± 0.71^d^	2.41 ± 0.04	10.23 ± 0.33^cd^
C200RM20	65.53 ± 0.60^abc^	16.48 ± 0.51^ab^	14.54 ± 0.24^bcd^	2.51 ± 0.13	9.85 ± 0.12^bcd^
CPRC20	66.27 ± 0.49^abc^	16.30 ± 0.18^ab^	13.26 ± 0.69^abcd^	2.56 ± 0.08	9.62 ± 0.20^abcd^
IRM20	65.78 ± 0.49^abc^	16.10 ± 0.38^ab^	14.23 ± 0.51^abcd^	2.61 ± 0.11	9.57 ± 0.16^abcd^
CRM20	65.04 ± 0.16^abc^	16.47 ± 0.56^ab^	14.47 ± 0.15^bcd^	2.53 ± 0.05	9.92 ± 0.05^bcd^
C95RM35	67.51 ± 1.18^bc^	15.08 ± 0.55^ab^	13.43 ± 0.74^abcd^	2.42 ± 0.13	9.26 ± 0.29^abc^
C200RM35	67.18 ± 1.16^bc^	16.32 ± 0.42^ab^	12.42 ± 0.47^ab^	2.56 ± 0.07	9.25 ± 0.29^abc^
CPRC35	68.46 ± 0.57^c^	15.80 ± 0.08^ab^	11.59 ± 0.47^a^	2.53 ± 0.09	8.84 ± 0.17^a^
IRM35	68.10 ± 0.18^c^	14.49 ± 0.49^a^	13.96 ± 0.70^b^	2.57 ± 0.13	9.07 ± 0.05^ab^
CRM35	66.28 ± 0.34^abc^	16.07 ± 0.56^ab^	14.34 ± 0.16^bcd^	2.56 ± 0.08	9.54 ± 0.08^abcd^

Values are means ± SEM (*n* = 3). Means in the same column with different superscripts are significantly different from each other (*p* < 0.05).

Dietary inclusion of 35% RSM did not affect the whole-body crude ash of rainbow trout (*p* > 0.05, Table 4). Dietary inclusion of 35% RSM regardless of sources generally increased the whole-body moisture content of fish, but reduced the whole-body crude protein, crude lipid, crude ash and total energy content, whereas no significant differences (*p* > 0.05) were observed in the whole-body moisture content between the CRM35 group and the control group, in the whole-body crude protein content between the C95RM35, C200RM35, CPRC35, or CRM35 groups and the control group, in the whole-body crude lipid content between the C95RM35 or CRM35 groups and the control group, in the whole-body crude ash content between the C95RM35, C200RM35, CPRC35, or CRM35 groups and the control group, in the whole-body total energy content between the CRM35 group and the control group.

With the increase of RSM inclusion level, the whole-body crude protein, crude lipid, crude ash and total energy content exhibited a descending trend, while the whole-body moisture showed an opposite trend (Table 4). However, no significant differences (*p* > 0.05) were observed in the whole-body moisture, crude protein, crude lipid, crude ash and total energy content among the C95RM, C200RM, CPRC, IRM, or CRM groups.

### 3.4 Digestive enzyme activity

Dietary inclusion of 20% RSM did not affect the intestinal disaccharidase activity (*p* > 0.05, Table 5). However, the inclusion of 20% RSM regardless of sources generally reduced the pepsin, intestinal lipase and amylase activities, whereas no significant differences (*p* > 0.05) were observed in the pepsin activity between the C95RM20, C200RM20, or CPRC20 groups and the control group, in the intestinal lipase activity between the C95RM20 or C200RM20 groups and the control group, in the intestinal amylase activity between the C200RM20 or CRM20 groups and the control group.

**Table 5:** Intestinal digestive enzymes activities of rainbow trout fed diets with different types and levels of rapeseed meal.

Diets	Pepsin (U/mg protein)	α-lipase (U/g protein)	Amylase (U/mg protein)	Disaccharidase (U/mg protein)
Control	13.40 ± 0.73^b^	22.09 ± 0.85^e^	0.51 ± 0.03^c^	9.17 ± 0.34^c^
C95RM20	9.38 ± 0.59^ab^	20.03 ± 1.67^de^	0.34 ± 0.03^ab^	6.98 ± 0.94^abc^
C200RM20	9.26 ± 1.31^ab^	18.15 ± 0.88^cde^	0.36 ± 0.05^abc^	9.95 ± 1.36^c^
CPRC20	9.72 ± 0.31^ab^	13.03 ± 1.98^abc^	0.35 ± 0.04^ab^	9.54 ± 0.41^c^
IRM20	7.83 ± 0.57^a^	10.39 ± 1.97^ab^	0.30 ± 0.03^ab^	7.27 ± 0.09^abc^
CRM20	8.58 ± 0.76^a^	15.61 ± 1.22^bcd^	0.42 ± 0.01^bc^	8.78 ± 0.56^bc^
C95RM35	8.36 ± 0.47^a^	18.69 ± 0.34^cde^	0.21 ± 0.02^a^	4.75 ± 0.49^a^
C200RM35	8.79 ± 1.06^a^	9.82 ± 1.26^ab^	0.22 ± 0.01^a^	7.36 ± 0.65^abc^
CPRC35	7.72 ± 1.72^a^	10.59 ± 1.93^ab^	0.24 ± 0.04^a^	7.04 ± 0.85^abc^
IRM35	6.99 ± 0.31^a^	8.74 ± 1.07^a^	0.27 ± 0.02^ab^	7.48 ± 0.53^abc^
CRM35	7.54 ± 0.95^a^	9.44 ± 0.42^ab^	0.22 ± 0.03^a^	5.55 ± 0.37^ab^

Values are means ± SEM (*n* = 3). Means in the same row with different superscripts are significantly different from each other (*p* < 0.05).

Dietary inclusion of 35% RSM regardless of sources generally reduced the pepsin, intestinal lipase, amylase and disaccharidase activities, whereas no significant differences (*p* > 0.05) were observed in the intestinal lipase activity between the C95RM35 group and the control group, in the intestinal disaccharidase activity between the C200RM35, CPRC35, or IRM35 groups and the control group (Table 5).

With the increase of RSM inclusion level, the digestive enzymes (pepsin, lipase, amylase, disaccharidase) activities exhibited a descending trend, but significant differences were only observed in the intestinal lipase activity among the C200RM groups, in the intestinal amylase activity among the CRM groups (*p* < 0.05, Table 5).

### 3.5 Protein metabolism-related parameters

Dietary inclusion of 20% RSM did not affect the serum GLU and TP content (*p* > 0.05, Table 6). Dietary inclusion of 20% RSM regardless of sources generally reduced the serum T_3_ and T_4_ content, but significant differences were only observed between the IRM20 or CPRC20 groups and the control group (*p* < 0.05). However, the inclusion of 20% RSM regardless of sources generally increased the serum TC, TG, and BUN content as well as AST and ALT activities, whereas no significant differences (*p* > 0.05) were observed in the serum TC content between the C200RM20, CPRC20, IRM20 or CRM20 groups and the control group, in the serum TG content between the C200RM20, IRM20, or CRM20 groups and the control group, in the serum AST and ALT activities between the C95RM20, C200RM20, or CRM20 groups and the control group.

**Table 6:** Plasma biochemical parameters of rainbow trout fed diets with different types and levels of rapeseed meal.

Diets	TP (g/L)	TC (mmol/L)	TG (mmol/L)	GLU (mmol/L)	BUN (mmol/L)	AST (IU/L)	ALT (IU/L)	T_3_ (ng/mL)	T_4_ (ng/mL)
Control	26.01 ± 1.79^ab^	15.88 ± 0.35^a^	1.94 ± 0.31^a^	3.38 ± 0.67^ab^	0.71 ± 0.02^a^	35.88 ± 6.00^a^	5.27 ± 0.29^a^	1.35 ± 0.03^d^	3.84 ± 0.20^e^
C95RM20	25.73 ± 0.72^a^	22.75 ± 0.68^b^	4.10 ± 0.50^bc^	3.85 ± 0.08^ab^	1.05 ± 0.02^bc^	62.58 ± 4.13^abc^	5.98 ± 0.51^ab^	1.25 ± 0.06^d^	3.52 ± 0.38^de^
C200RM20	26.44 ± 0.12^ab^	18.05 ± 1.12^ab^	2.49 ± 0.09^ab^	2.95 ± 0.69^ab^	1.12 ± 0.03^bcd^	48.86 ± 6.12^ab^	5.62 ± 0.42^a^	1.20 ± 0.03^cd^	3.89 ± 0.12^e^
CPRC20	29.93 ± 1.19^b^	18.05 ± 1.61^ab^	4.41 ± 0.19^bc^	3.28 ± 0.37^ab^	1.20 ± 0.03^def^	92.50 ± 6.07^cde^	9.56 ± 0.39^cde^	0.94 ± 0.03^bc^	2.19 ± 0.19^ab^
IRM20	27.86 ± 0.19^ab^	18.71 ± 1.54^ab^	3.60 ± 0.49^ab^	4.83 ± 0.47^b^	1.24 ± 0.02^def^	95.54 ± 4.13^de^	9.12 ± 0.38^bcde^	0.87 ± 0.06^b^	2.38 ± 0.18^abc^
CRM20	26.80 ± 0.29^ab^	15.75 ± 0.65^a^	3.13 ± 0.45^ab^	2.94 ± 0.14^ab^	1.03 ± 0.04^b^	47.75 ± 6.05^ab^	6.59 ± 0.61^abc^	1.34 ± 0.10^d^	3.37 ± 0.21^cde^
C95RM35	26.23 ± 0.71^ab^	20.05 ± 0.16^ab^	3.16 ± 0.39^ab^	2.90 ± 0.54^ab^	1.29 ± 0.04^efg^	77.75 ± 8.02^bcde^	7.10 ± 0.68^abcd^	0.88 ± 0.08^b^	2.49 ± 0.22^bcd^
C200RM35	27.87 ± 0.61^ab^	21.31 ± 0.65^ab^	3.74 ± 0.41^abc^	3.13 ± 0.13^ab^	1.20 ± 0.01^cdef^	64.70 ± 3.51^abcd^	7.34 ± 0.46^abcd^	0.95 ± 0.03^bc^	2.44 ± 0.29^abc^
CPRC35	29.08 ± 0.56^ab^	17.92 ± 1.66^ab^	5.81 ± 0.57^c^	2.59 ± 0.35^a^	1.41 ± 0.06^g^	91.67 ± 8.36^cde^	10.03 ± 0.81^de^	0.31 ± 0.03^a^	1.42 ± 0.12^a^
IRM35	26.30 ± 0.37^ab^	18.74 ± 1.70^ab^	4.33 ± 0.48^bc^	4.23 ± 0.22^ab^	1.35 ± 0.03^fg^	106.51 ± 5.76^e^	12.12 ± 1.22^e^	0.30 ± 0.05^a^	1.44 ± 0.20^ab^
CRM35	28.22 ± 0.62^ab^	19.19 ± 0.85^ab^	2.62 ± 0.50^ab^	2.59 ± 0.17^a^	1.15 ± 0.01^bcde^	66.31 ± 7.24^abcd^	10.94 ± 0.58^e^	0.82 ± 0.07^b^	1.88 ± 0.05^ab^

Values are means ± SEM (*n* = 3). Means in the same row with different superscripts are significantly different from each other (*p* < 0.05). TP, total protein; TC, total cholesterol; TG, triglyceride; GLU, glucose; BUN, blood urea nitrogen; AST, aspartate aminotransferase; ALT, lanine aminotransferase; T_3_, triiodothyronine; T_4_, thyroxine.

Dietary inclusion of 35% RSM did not affect the serum TP, TC, and GLU content (*p* > 0.05, Table 6). Dietary inclusion of 35% RSM significantly reduced the serum T_3_ and T_4_ content (*p* < 0.05). However, the inclusion of 35% RSM regardless of sources generally increased the serum TG, BUN, AST, and ALT content/activities, whereas no significant differences (*p* > 0.05) were observed in the serum TG content between the CPRC35 or IRM35 groups and the control group, in the serum AST activity between the C200RM35 or CRM35 groups and the control group, in the serum ALT activity between the C95RM35 or C200RM35 groups and the control group.

With the increase of RSM inclusion level, the protein metabolism-related indexes (TP, TC, TG, BUN, AST, and ALT) exhibited a incrementing trend but the serum T_3_ and T_4_ levels showed the opposite trend (Table 6). However, significant differences were only observed in the serum BUN and T_3_ content among the C95RM or CPRC groups, in the serum T_4_ content among the C200RM groups, in the serum T_3_ content among the IRM groups, in the serum ALT, T_3_ and T_4_ activities/content among the IRM groups (*p* < 0.05).

### 3.6 Antioxidant-related parameters

Dietary inclusion of 20% RSM did not affect the plasma SOD, GR, and AKP as well as hepatic SOD, POD, GR, and GPx activities (*p* > 0.05, Tables 7, 8). Dietary inclusion of 20% RSM regardless of sources generally reduced the plasma or hepatic CAT, POD, and GPx activities, whereas no significant differences (*p* > 0.05) were observed in the plasma CAT activity between the IRM20 or CRM20 groups and the control group, in the hepatic CAT activity between the C95RM20, C200RM20, or CRM20 groups and the control group, in the plasma POD activity between the C95RM20, C200RM20, IRM20, or CRM20 groups and the control group, in the plasma GPx activity between the C95RM20, C200RM20, or CRM20 groups and the control group. In contrast, dietary inclusion of 20% RSM regardless of sources generally increased the plasma cortisol and MDA content, whereas no significant differences (*p* > 0.05) were observed in the plasma cortisol and MDA content between the C95RM20, C200RM20, or CRM20 groups and the control group, in the hepatic MDA content between the CRM20 group and the control group.

**Table 7:** Antioxidant-related index in plasma of rainbow trout fed diets with different types and levels of rapeseed meal.

Diets	SOD (U/mL)	CAT (U/mL)	POD (U/mL)	GPx (U/μL)	GR (U/L)	Cortisol (ng/mL)	MDA (nmol/mL)
Control	21.49 ± 0.20	19.19 ± 1.24^d^	30.45 ± 1.34^e^	0.22 ± 0.00^d^	5,359 ± 5.67	0.10 ± 0.00^a^	4.70 ± 0.96^a^
C95RM20	20.98 ± 0.44	14.84 ± 0.41^abc^	28.22 ± 1.05^de^	0.21 ± 0.00^cd^	55.74 ± 5.67	0.12 ± 0.01^ab^	6.92 ± 1.12^a^
C200RM20	20.59 ± 0.54	15.33 ± 0.66^bc^	31.27 ± 0.60^e^	0.19 ± 0.00^bcd^	70.74 ± 9.86	0.09 ± 0.00^a^	11.20 ± 1.48^ab^
CPRC20	21.61 ± 0.42	14.96 ± 0.40^abc^	21.85 ± 1.25^abc^	0.17 ± 0.01^ab^	57.88 ± 6.43	0.21 ± 0.01^cd^	17.26 ± 0.99^bc^
IRM20	21.95 ± 0.49	16.56 ± 0.71^cd^	26.44 ± 0.90^cde^	0.16 ± 0.01^ab^	62.17 ± 8.57	0.20 ± 0.02^cd^	30.52 ± 2.09^d^
CRM20	21.49 ± 0.32	16.44 ± 1.18^cd^	30.59 ± 1.16^e^	0.21 ± 0.00^cd^	68.60 ± 5.67	0.14 ± 0.02^abc^	13.08 ± 0.90^abc^
C95RM35	21.33 ± 0.51	12.65 ± 0.32^ab^	22.59 ± 0.91^abc^	0.19 ± 0.00^bcd^	75.03 ± 5.67	0.18 ± 0.01^bcd^	10.77 ± 0.51^ab^
C200RM35	21.91 ± 0.24	12.77 ± 0.39^ab^	23.63 ± 0.39^bcd^	0.19 ± 0.00^bcd^	47.16 ± 2.14	0.19 ± 0.01^cd^	14.53 ± 2.27^abc^
CPRC35	20.82 ± 0.39	11.88 ± 0.64^ab^	19.33 ± 1.60^ab^	0.14 ± 0.01^a^	68.60 ± 2.14	0.24 ± 0.02^d^	21.20 ± 4.15^cd^
IRM35	20.13 ± 0.38	11.53 ± 0.61^a^	18.00 ± 1.22^a^	0.14 ± 0.01^a^	49.31 ± 5.67	0.32 ± 0.02^e^	46.67 ± 2.57^e^
CRM35	20.70 ± 0.31	13.85 ± 0.40^abc^	21.56 ± 0.80^abc^	0.18 ± 0.01^bc^	53.59 ± 2.14	0.18 ± 0.01^bcd^	21.67 ± 1.48^cd^

Values are means ± SEM (*n* = 3). Means in the same row with different superscripts are significantly different from each other (*p* < 0.05). SOD, superoxide dismutase; CAT, catalase; POD, peroxidase; GPx, glutathione peroxidase; GR, glutathione reductase; MDA, malondialdehyde.

**Table 8:** Antioxidant-related index in liver of rainbow trout fed diets with different types and levels of rapeseed meal.

Diets	SOD (U/mg protein)	CAT (U/mg protein)	POD (U/mg protein)	GR (U/g protein)	GPx (U/μg protein)	MDA (nmol/mg protein)
Control	21.77 ± 0.13	20.93 ± 2.44^c^	26.88 ± 0.37	72.89 ± 3.21	17.28 ± 1.37	4.16 ± 0.42^a^
C95RM20	22.18 ± 0.31	16.63 ± 0.36^abc^	23.93 ± 2.57	82.51 ± 5.31	12.96 ± 0.33	9.07 ± 0.33^bc^
C200RM20	22.88 ± 0.07	18.25 ± 1.76^bc^	28.91 ± 3.56	86.82 ± 4.32	16.75 ± 2.21	9.91 ± 0.68^bcd^
CPRC20	22.25 ± 0.27	13.91 ± 1.81^ab^	21.92 ± 2.41	66.21 ± 3.70	14.41 ± 2.64	10.28 ± 1.05^bcd^
IRM20	21.77 ± 0.14	12.32 ± 0.36^ab^	21.95 ± 1.30	83.27 ± 8.02	16.31 ± 1.14	13.24 ± 0.56^cde^
CRM20	22.96 ± 0.48	18.73 ± 1.20^bc^	27.07 ± 1.97	73.68 ± 8.32	19.69 ± 2.03	6.30 ± 1.09^ab^
C95RM35	22.54 ± 0.44	14.21 ± 1.27^abc^	23.05 ± 1.36	78.09 ± 9.84	12.42 ± 1.30	11.95 ± 0.56^cde^
C200RM35	21.86 ± 0.34	13.07 ± 1.31^ab^	23.94 ± 2.78	87.04 ± 6.40	15.18 ± 1.63	13.33 ± 0.64^cde^
CPRC35	21.84 ± 0.17	14.14 ± 1.48^abc^	23.76 ± 1.79	87.88 ± 5.95	14.53 ± 1.36	13.71 ± 1.58^de^
IRM35	22.50 ± 0.49	11.23 ± 0.68^a^	20.25 ± 2.96	77.31 ± 4.99	15.78 ± 1.99	16.30 ± 1.45^e^
CRM35	23.10 ± 0.19	16.82 ± 0.19^abc^	20.35 ± 1.11	63.33 ± 5.43	14.82 ± 0.99	9.45 ± 0.58^bcd^

Values are means ± SEM (*n* = 3). Means in the same row with different superscripts are significantly different from each other (*p* < 0.05). SOD, superoxide dismutase; CAT, catalase; POD, peroxidase; GR, glutathione reductase; GPx, glutathione peroxidase; MDA, malondialdehyde.

Dietary 35% RSM inclusion regardless of sources had no significant effects on the plasma SOD, GR, and AKP activities as well as hepatic SOD, POD, GR, and GPx activities (*p* > 0.05, Tables 7, 8). Dietary inclusion of 35% RSM regardless of sources generally reduced the plasma CAT, POD and GPx activities, whereas no significant differences (*p* > 0.05) were observed in the hepatic CAT activity between the C95RM35, CRM35, or CPRC35 groups and the control group, in the plasma GPx activity between the C95RM35 or C200RM35 groups and the control group. In contrast, dietary inclusion of 35% RSM regardless of sources generally increased the plasma cortisol and MDA content, whereas no significant differences (*p* > 0.05) were observed in the plasma MDA content between the C95RM35 or C200RM35 groups and the control group.

With the increase of RSM inclusion level, the antioxidant-related enzymes (SOD, CAT, POD, GPx, and AKP) activities exhibited a descending trend and the plasma cortisol and MDA content showed the opposite trend (Tables 7, 8). However, significant differences were only observed in the plasma POD activity among the C95RM, C200RM, IRM, or CRM groups, in the plasma cortisol content among the C200RM or IRM groups, in the plasma CAT activity and MDA content among the IRM groups (*p* < 0.05).

### 3.7 Non-specific immunity response

Dietary inclusion of 20% RSM did not affect the serum IgM and C4 content as well as PA of head kidney macrophages (*p* > 0.05, Table 9). Dietary inclusion of 20% RSM regardless of sources generally reduced the serum C3 content, the serum and intestinal LZM activity, and the RBA of head kidney macrophages, whereas no significant differences (*p* > 0.05) were observed in the serum C3 content and LZM activity between the C95RM20, C200RM20, or CRM20 groups and the control group, in the intestinal LZM activity among the C95RM20, C200RM20 CPRC20, or CRM20 groups and the control group, in the RBA of head kidney macrophages among the C200RM20 group and the control group.

**Table 9:** Immune response and disease resistance of rainbow trout fed diets with different types and levels of rapeseed meal.

Diets	IgM (g/L)	C3 (g/L)	C4 (g/L)	LZM (μg/mL)	LZM (μg/mg protein)	AKP (U/dL)	RBA	PA
Control	1.63 ± 0.08^de^	0.85 ± 0.03^e^	0.35 ± 0.01	4.37 ± 0.12^g^	84.42 ± 7.25^c^	11.74 ± 1.52	2.20 ± 0.05^g^	0.57 ± 0.01^cde^
C95RM20	1.46 ± 0.08^cde^	0.80 ± 0.03^e^	0.37 ± 0.01	3.72 ± 0.03^efg^	66.33 ± 4.18^bc^	12.36 ± 1.03	2.05 ± 0.03^ef^	0.58 ± 0.01^cde^
C200RM20	1.77 ± 0.07^e^	0.84 ± 0.03^e^	0.37 ± 0.01	3.51 ± 0.20^defg^	58.96 ± 6.80^abc^	13.47 ± 1.42	2.10 ± 0.04^fg^	0.62 ± 0.02^de^
CPRC20	1.34 ± 0.06^bcd^	0.67 ± 0.03^abc^	0.39 ± 0.01	2.95 ± 0.17^bcde^	51.59 ± 7.73^abc^	15.68 ± 1.16	1.73 ± 0.04^b^	0.51 ± 0.02^abc^
IRM20	1.31 ± 0.02^abcd^	0.75 ± 0.01^bcd^	0.40 ± 0.01	3.37 ± 0.09^cdef^	36.18 ± 2.32^ab^	15.15 ± 1.19	1.69 ± 0.01^ab^	0.56 ± 0.01^bcde^
CRM20	1.60 ± 0.12^de^	0.86 ± 0.04^e^	0.39 ± 0.03	3.92 ± 0.30^fg^	82.41 ± 9.92^c^	14.32 ± 0.44	1.92 ± 0.02^de^	0.63 ± 0.02^e^
C95RM35	1.12 ± 0.05^ab^	0.75 ± 0.01^bcd^	0.40 ± 0.01	2.43 ± 0.07^b^	42.97 ± 7.46^ab^	12.73 ± 0.18	1.90 ± 0.03^cd^	0.56 ± 0.01^bcde^
C200RM35	1.04 ± 0.04^ab^	0.82 ± 0.02^e^	0.41 ± 0.03	2.60 ± 0.10^bc^	42.88 ± 9.87^ab^	12.63 ± 0.72	1.90 ± 0.02^cd^	0.50 ± 0.02^ab^
CPRC35	1.02 ± 0.01^a^	0.65 ± 0.04^ab^	0.36 ± 0.01	2.74 ± 0.19^bcd^	36.27 ± 7.61^ab^	12.42 ± 0.89	1.56 ± 0.01^a^	0.46 ± 0.01^a^
IRM35	1.00 ± 0.03^a^	0.60 ± 0.03^a^	0.42 ± 0.02	1.15 ± 0.18^a^	24.12 ± 3.07^a^	14.89 ± 0.76	1.66 ± 0.01^ab^	0.52 ± 0.01^abc^
CRM35	1.23 ± 0.01^abc^	0.81 ± 0.02^e^	0.41 ± 0.03	3.51 ± 0.25^defg^	46.23 ± 7.25^ab^	10.81 ± 0.47	1.77 ± 0.04^bc^	0.55 ± 0.01^bcd^

Values are means ± SEM (*n* = 3). Means in the same row with different superscripts are significantly different from each other (*p* < 0.05). IgM, immunoglobulin M; C3, complement 3; C4, complement 4; LZM, lysozyme; AKP, alkaline phosphatase; RBA, respiratory burst activity of head kidney macrophages; PA, phagocytic activity of head kidney macrophages.

Dietary inclusion of 35% RSM significantly reduced the serum IgM and intestinal LZM activities as well as RBA of head kidney macrophages (*p* < 0.05), while had no significant effect on the serum C4 content and AKP activity (*p* > 0.05, Table 9). Dietary inclusion of 35% RSM regardless of sources generally reduced the serum C3 content and LZM activity as well as PA of head kidney macrophages, whereas no significant differences (*p* > 0.05) were observed in the serum C3 content between the C200RM35 or CRM35 groups and the control group, in the serum LZM activity between the CRM35 group and the control group, in the PA of head kidney macrophages between the C95RM35, IRM35, or CRM35 groups and the control group.

With the increase of RSM inclusion level, the non-specific immune-related parameters (the serum IgM, C3 and LZM content/activities, the intestinal LZM activity, the RBA and PA of head kidney macrophages) exhibited a descending trend (Table 9). However, significant differences were only observed in the serum IgM content and RBA of head kidney macrophages among the C95RM, C200RM, CPRC, or CRM groups, in the serum C3 content among the C95RM or IRM groups, in the PA of head kidney macrophages among the C200RM or CRM groups, in the serum LZM activity among the C95RM, C200RM, or IRM groups, in the intestinal LZM activity among the CRM group (*p* < 0.05).

### 3.8 Pathogenic challenge

The survival rate and RPS of rainbow trout challenged with *A. hydrophila* were shown in Table 10. Dietary RSM inclusion regardless of source and level generally depressed the survival rate, which varied from 25 to 60%. The RPS was significantly depressed by the inclusion of C200RM20 (*p* < 0.05) and CPRC20 (*p* < 0.05), highly significantly depressed by the inclusion of IRM20 (*p* < 0.01), C95RM35 (*p* < 0.01), C200RM35 (*p* < 0.001), CPRC35 (*p* < 0.001), and IRM35 (*p* < 0.001).

**Table 10:** Relative percentage survival (RPS) of rainbow trout after infection with *Aeromonas hydrophila*.

Diets	Number of challenged fish	Number of dead fish (mortality, %)	Survival (%)	RPS (%)
Control	40	14	65	–
C95RM20	40	18	55	−28.57
C200RM20	40	20	50	−42.86*
CPRC20	40	20	50	−42.86*
IRM20	40	24	40	−71.43**
CRM20	40	16	60	−14.29
C95RM35	40	24	40	−71.43**
C200RM35	40	26	35	−85.71***
CPRC35	40	26	35	−85.71***
IRM35	40	30	25	−114.29***
CRM35	40	16	60	−14.29

*p < 0.05; **p < 0.01; ***p < 0.001.

### 3.9 Histomorphology of liver

**Figure 1:**
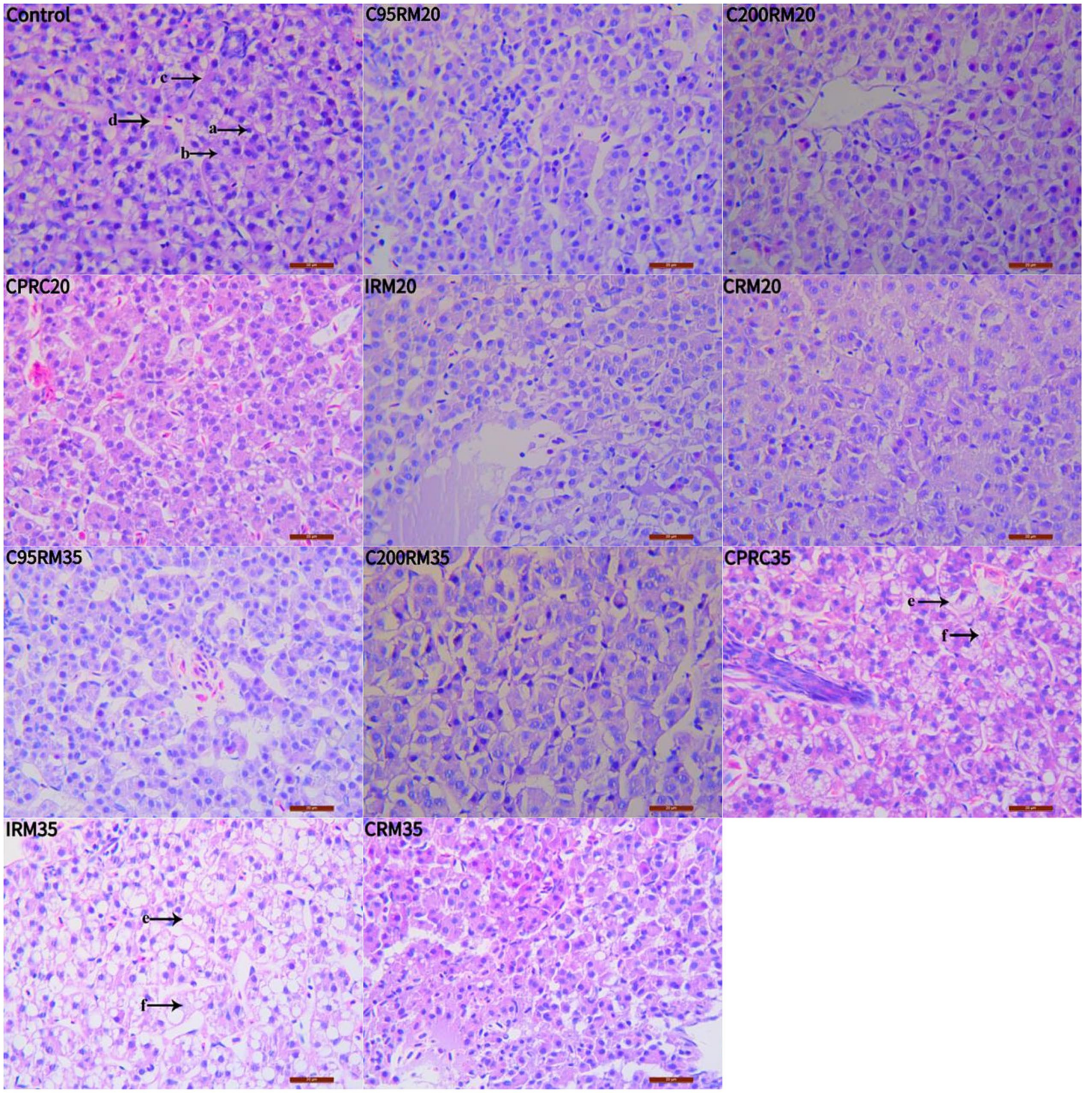
Effects of different types and levels of rapeseed meal on liver tissue of rainbow trout (400×; H&E stain). The letters in the image indicate: a, nucleus; b, cytoplasm; c, cell membrane; d, hepatic blood sinusoids; e, cell vacuolization; f, nucleus deviation.

**Figure 2:**
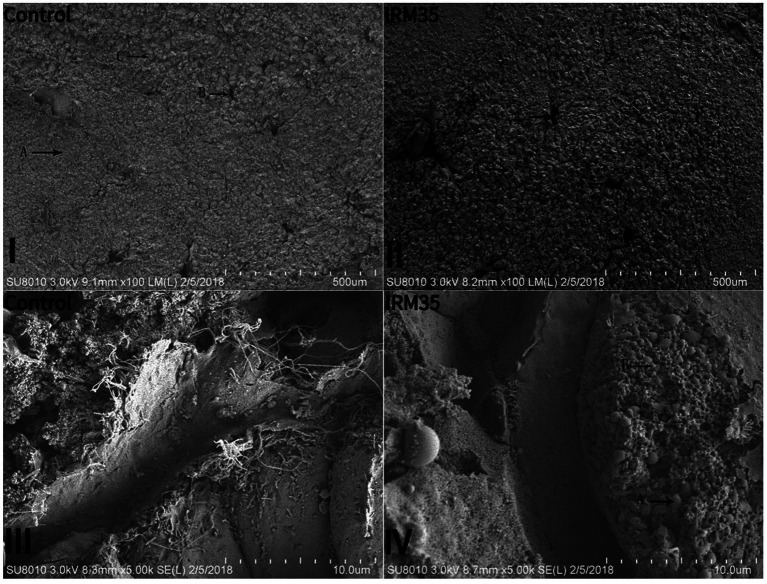
Effect of different types and levels of rapeseed meal on the liver tissue of rainbow trout. **(I,II)** Histomorphology of the liver under the scanning electron microscope (1,000×). A, liver peritoneum; B, hepatic sinusoids; C, liver cell. **(III,IV)** Histomorphology of the liver under the scanning electron microscope (5,000×). A, lipid droplet; B, mitochondria.

Hepatocyte vacuolation and nuclear migration were observed in the livers of fish fed CPRC and IRM diets, and the severity of these effects increased with higher levels of CPRC and IRM inclusion (Figure 1). Fish fed the IRM35 diet showed more severe liver damage, including liver envelope damage (Figures 2I,II, arrow A), liver sinus atrophy (Figures 2I,II, arrow B), increased accumulation of lipid droplets (Figures 2III,IV, arrow A) and mitochondrial damage (Figures 2III,IV, arrow B).

### 3.10 Histomorphology of intestine

**Figure 3:**
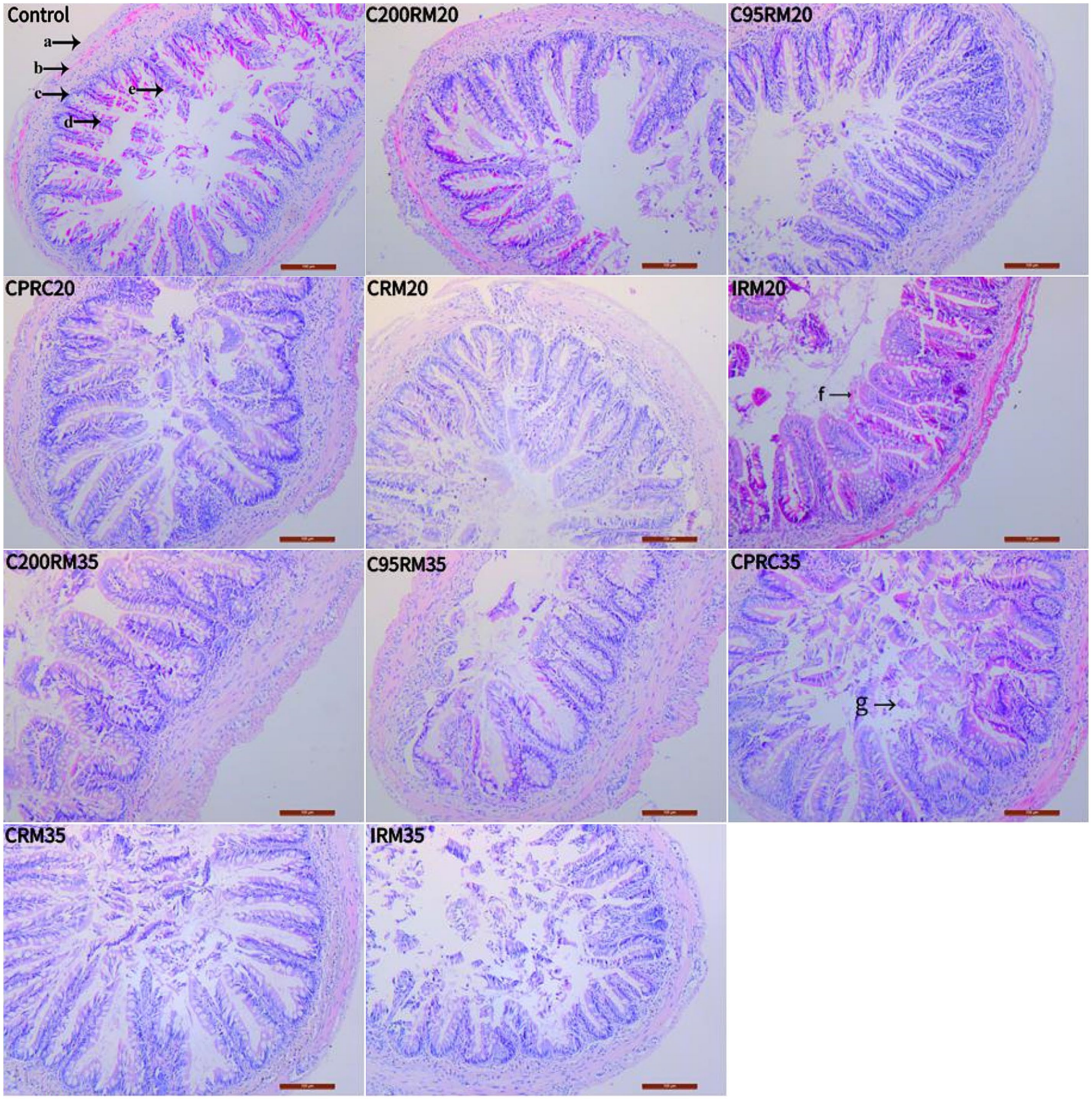
Effect of different types and levels of rapeseed meal on the intestinal tissues of rainbow trout (100×; H&E stain). The letters in the image indicate: a, intestinal plasma membrane; b, muscle layer; c, submucosa; d, mucosa layer; e, cupped cells (e); f, villi blunting; g, villi.

**Figure 4:**
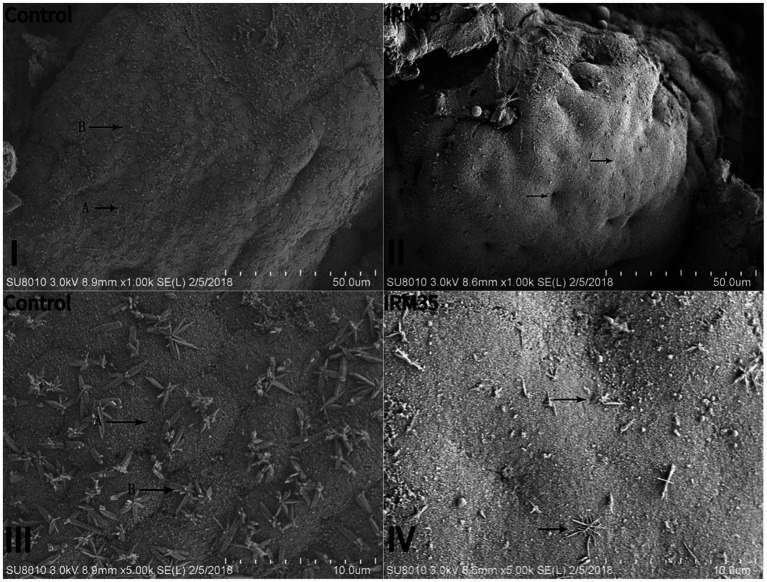
Effects of different types and levels of rapeseed meal on intestinal tissue of rainbow trout. **(I,II)** Histomorphology of the intestinal under the scanning electron microscope (1,000×). **(III,IV)** Histomorphology of the intestinal under the scanning electron microscope (5,000×). A, columnar epithelial cell; B, intestinal villi.

Fish fed the five RSM diets exhibited varying degrees of goblet hyperplasia (Figure 3, arrow e) and villi loss or erosion (Figure 3, arrow g) in their intestines. Fish fed CPRC and IRM diets experienced more severe intestinal damage. At the same time, fish fed the IRM35 diet exhibited the most severe intestinal damage, which included blurred or disappeared boundaries of intestinal cells, a significantly reduced number of columnar epithelial cells (Figure 4, arrow A), and severe damage to intestinal villi (Figure 4, arrow B).

### 3.11 Histomorphology of gill

There was no significant damage to the gills of rainbow trout fed the five RSM diets. Only enlargement or an increase in thyroid follicles was observed (Supplementary Figure 1).

## 4 Discussion

The nutritional quality of RSM is influenced by various factors, including the source of RSM, the processing technology, and the content of ANFs (10, 15, 24). But much depends on the level of ANFs, which negatively affect digestive enzymes’ activities, nutrients’ digestibility, and the growth rate of fish (10). Among them, the glucosinolates had the most negative effect (25). In this experiment, the glucosinolates content from high to low was CPRC (6.56%), IRM (4.93%), C200RM (0.97%), C95RM (0.60%), and CRM (not detected). Depending on the processing method, the nutritional value of RSM produced by the pre-pressure extraction method (CRM and C200RM) is generally higher than that produced by the direct pressing method (C95RM), low temperature direct pressing method (CPRC) and press cold soaking method (IRM) (26–28). In addition, according to our test results, the ANFs content of IRM was higher than that of CPRC and the amino acid was unbalanced. In summary, the nutritional quality of the five RSM was about CRM, C200RM, C95RM, CPRC, and IRM from best to worst.

Burel et al. (25) reported the adverse effects of four levels of glucosinolates (1.4, 2.3, 11.6, 19.35, and 41.0 mol/kg) in RSM (low glucosinolates content) on the growth and dry matter of rainbow trout. In the present study, diets containing ≥20% RSM significantly reduced the growth rate of rainbow trout. The results were similar in barramundi (*Lates calcarifer*) (30% RSM) (29) and Ussuri catfish (20% RSM) (30). However, Cui et al. (31) reported that rainbow trout can tolerate up to 30% RSM, which could be attributed to variations in fish species, sizes, or dietary composition (2). These results suggest that a high level of dietary RSM reduces the growth rate of rainbow trout, and its harmful effects may be primarily associated with glucosinolates in ANFs. Thiocyanates, isothiocyanates (the first two are degradation products of glucosinolates), phytic acid, and tannins interfere with the digestion and absorption of nutrients. Additionally, they have a toxic effect on the thyroid gland, but this is only true for thiocyanates and isothiocyanates (25, 32). The presence of glucosinolates in RSM in the diet is considered to be the main cause of reduced growth performance in fish (10, 15).

The intestine is the primary organ responsible for digestion and absorption in fish, and its digestive enzymes in the intestine play a crucial role in the digestion and absorption of nutrients. Furthermore, the health of the intestine and the activities of digestive enzymes directly or indirectly impact fish growth (33, 34). In a previous study, it was found that the inclusion level of RSM at ≥17.2% in Japanese seabass (*Lateolabrax japonicus*) diets resulted in a reduction of protease, lipase, and α-amylase (35). In the present study, all groups with dietary inclusion of 20% RSM had varying degrees of intestinal damage and significant reductions in intestinal protease, lipase, or α-amylase activities, suggesting that the presence of ≥20% RSM in rainbow trout diets negatively affects intestinal health as well as intestinal digestive enzyme activities. This is closely related to the presence of ANFs (e.g., glucosinolates, phytic acid, tannins, and fiber) in RSM, which irritate the digestive mucosa (glucosinolates) and reduce digestive enzyme (protease, lipase and amylase) activities, thereby decreasing nutrient digestibility (36–38).

Blood parameters reflect changes in the metabolism of substances and the functional status of tissues or organs in the body. Among these parameters, the activities of ALT and AST are often used to assess liver health in fish (38, 39). The present study observed that the AST or ALT activity indicated an increased risk of liver injury when consuming a diet containing ≥20% RSM: this was supported by histological analyses of the liver. This is similar to the results of dietary RSM in grass carp (14), gibel carp (40), and Nile tilapia (41). Previous studies have shown that ANFs (glucosinolates and its degradation products) may play a significant role in forming liver lesions or injuries in aquatic animals when they consume specific amounts of RSM (42, 43). Meanwhile, Chen et al. (19) verified that allyl isothiocyanate (a degradation product of glucosinolates) was responsible for damaging the structure of hepatocytes using *in vitro* cellular analysis. The significant increase in plasma BUN levels in rainbow trout in all groups of dietary RSM suggests that dietary RSM may promote proteolytic metabolism (34). This may be related to the reduced intake of carbohydrates and lipids, resulting in greater utilization of proteins for catabolism to provide energy, which could also explain the decreased crude protein content of the fish. In addition, T_3_ and T_4_ are two forms of thyroid hormones that reflect the body’s metabolism of proteins, fats, and sugars. All four products of thioglucoside are toxic to fish. One of them, oxazolidinethione, is able to impede the synthesis of thyroxine and cause an increase in thyrotropin secretion from the pituitary gland, leading to goiter. In addition, isothiocyanate can compete with iodine to enter the thyroid gland, which will correspondingly reduce the uptake of iodine by the thyroid gland, thus causing goiter (16). In the present study, the levels of T_3_ and T_4_ were significantly decreased in the CPRC20, IRM20, C95RM35, C200R35, and CRM35 groups. This finding is consistent with the results reported by Danwitz et al. (44), where serum T_3_ levels tended to decrease with increasing levels of glucosinolates. Therefore, it can be inferred that glucosinolates may have a negative impact on thyroid function in fish, consequently affecting substance metabolism and tissue health.

The organism’s ability to generate and scavenge free radicals is normally in a dynamic equilibrium. However, when this equilibrium is disrupted, it leads to oxidative stress in the organism (34). Among them, antioxidant enzymes (e.g., GPx, POD, and CAT) are considered to be important defense mechanisms of the organism against free radicals (45). MDA, the end product of lipid peroxidation, is commonly used as a biological marker to measure oxidative stress (46). In the present study, it was observed that when the inclusion level of RSM was ≥20%, there was a decrease in plasma GPx, POD and CAT activities to varying degrees. Additionally, there was a significant increase in plasma cortisol and MDA levels. The above results suggest that high levels of RSM reduce antioxidant capacity and increase oxidative stress in fish, which is consistent with previously reported findings (30, 47). In addition, it has been suggested that the presence of ANFs in RSM may act as a potential stressor, leading to oxidative stress in fish and accumulating adverse effects that inhibit growth (48). It has been found that dietary CPRC and IRM in carp are more likely to cause a significant reduction in serum CAT levels, and there is a relationship with their glucosinolates, isothiocyanate, and oxazolidinethione levels (15). Therefore, it can be inferred that these ANFs may be thioglucosides and their hydrolysis products.

The non-specific immune system plays an important role in disease resistance in fish (49), whereas LZM, C3, and C4 play important roles in the immune system (50), with C3 and C4 being involved in a variety of immune responses. Changes in their levels are typically associated with inflammatory responses and immune system activity. Under the conditions of this assay, a dietary inclusion level of 20% RSM resulted in a general decrease in C3 and LZM activity. The activity significantly decreased as the levels of RSM inclusion increased. The same results were found in *Pseudobagrus ussuriensis* (30, 51, 52). In addition, the intestine is one of the important immune organs in fish (53). The presence of glucosinolates and its hydrolysis products in RSM can irritate the intestinal tract, damage the structure of the intestinal mucosa, inhibit the digestion and absorption of nutrients, and also affect the function of the fish body’s thyroid gland and liver health (54). These ultimately lead to a decrease in the fish’s immunity. Therefore, the results of the present experiment indicate that incorporating ≥20% RSM in the diet negatively impacted the non-specific immunity of rainbow trout.

## 5 Conclusion

The inclusion level of C200RM, C95RM, CPRC, IRM, or CRM in diets for rainbow trout should be less than 20%, otherwise had negative impacts on the growth performance, antioxidant capacity, immunity and health status of rainbow trout. In descending order, the utilization efficiency of RSM was CRM > C200RM > C95RM > CPRC > IRM, which may be due to the presence of anti-nutritional factors (mainly glucosinolates). A complete dosage study is also needed to find optimal levels of RSM in rainbow trout diets of different size ranges.

## Data availability statement

The original contributions presented in the study are included in the article/supplementary material, further inquiries can be directed to the corresponding authors.

## Ethics statement

The animal study was approved by Animal Research and Ethics Committee of Guangdong Ocean University. The study was conducted in accordance with the local legislation and institutional requirements.

## Author contributions

WJ: Writing – original draft, Data curation. HW: Data curation, Writing – original draft. LZ: Conceptualization, Writing – review & editing, Funding acquisition, Supervision. HM: Funding acquisition, Writing – review & editing, Supervision. JD: Writing – review & editing, Funding acquisition, Project administration, Supervision.
