# Supplementary material for: High replacement of soybean meal by different types of rapeseed meal is detrimental to rainbow trout (Oncorhynchus mykiss) growth, antioxidant capacity, non-specific immunity and Aeromonas hydrophila tolerance
Source: Front Nutr. 2024 Feb 6;11:1363411. doi: 10.3389/fnut.2024.1363411 (PMC10876845; doi:10.3389/fnut.2024.1363411)
Supplement: Supplementary file 1 [file Data_Sheet_1.docx]

## 1 Supplementary Figures


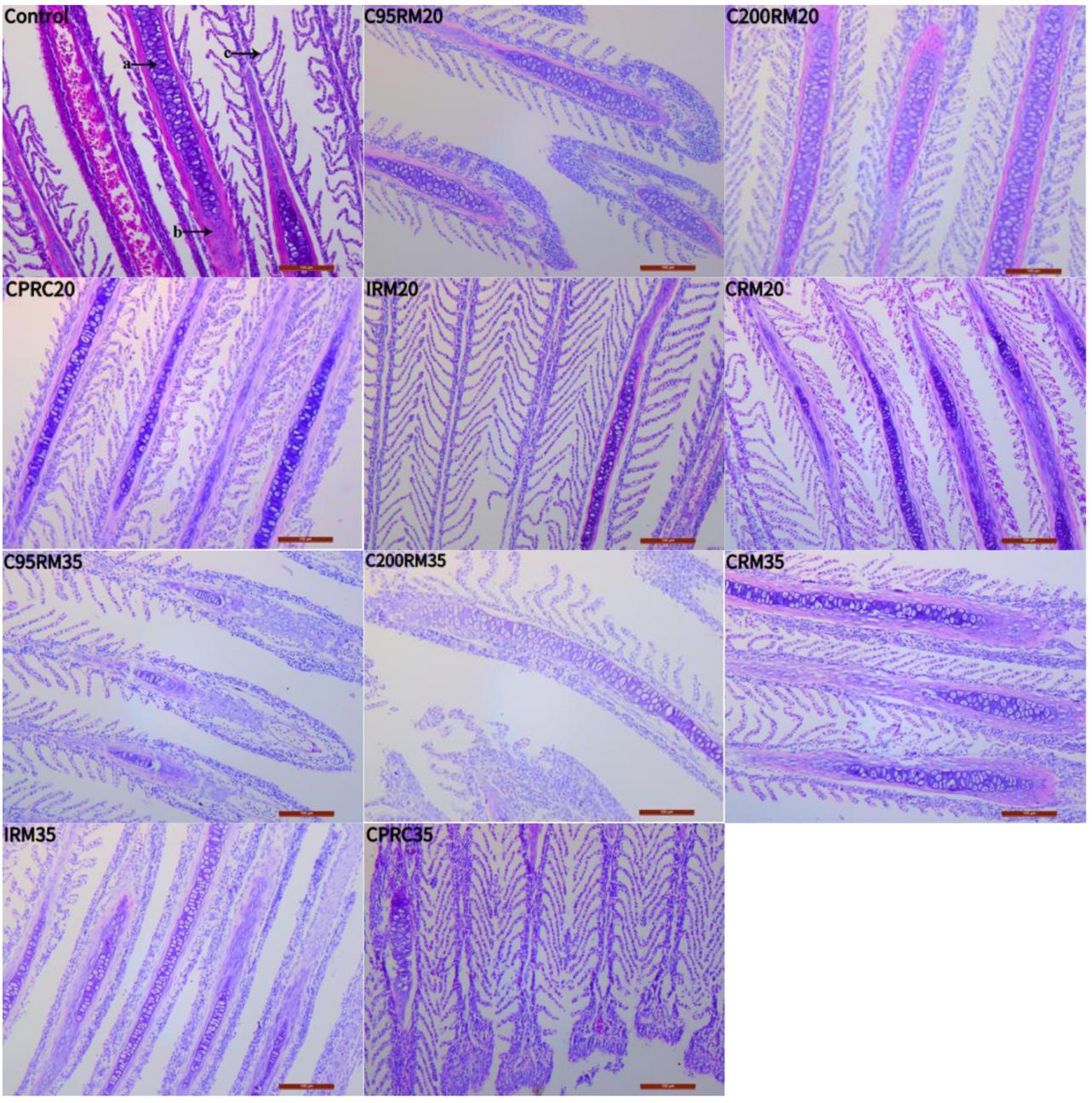


**Supplementary Figure 1.** Effect of different types and levels of rapeseed meal on gill tissue of rainbow trout (100×; H&E stain). The letters in the image indicate: a, thyroid follicle; b, gill filament; c, gill lamellae.
